# Effect of Mydriasis-Caused Intraocular Pressure Changes on Corneal Biomechanical Metrics

**DOI:** 10.3389/fbioe.2021.751628

**Published:** 2021-11-26

**Authors:** Yufeng Ye, Yi Li, Zehui Zhu, Anas Ziad Masoud Abu Said, Kevin Nguelemo Mayopa, Stephen Akiti, Chengyi Huang, Bernardo T. Lopes, Ashkan Eliasy, Yuanyuan Miao, Junjie Wang, Xiaobo Zheng, Shihao Chen, Fangjun Bao, Ahmed Elsheikh

**Affiliations:** ^1^ Eye Hospital, Wenzhou Medical University, Wenzhou, China; ^2^ North Huashan Hospital, Fudan University, Shanghai, China; ^3^ School of Engineering, University of Liverpool, Liverpool, United Kingdom; ^4^ The Institute of Ocular Biomechanics, Wenzhou Medical University, Wenzhou, China; ^5^ National Institute for Health Research (NIHR), Biomedical Research Centre for Ophthalmology, Moorfields Eye Hospital NHS Foundation Trust, UCL Institute of Ophthalmology, London, United Kingdom; ^6^ Beijing Advanced Innovation Center for Biomedical Engineering, Beihang University, Beijing, China

**Keywords:** biomechanical metrics, intraocular pressure, mydriasis, stiffness parameter at first applanation, stress-strain index

## Abstract

**Purpose:** To evaluate the dependence of biomechanical metrics on intraocular pressure (IOP).

**Methods:** 233 refractive surgery patients were included in this study—all were examined 3 times with the Corvis ST before and after dilation, and the differences (∆) in the main device parameters were assessed. The data collected included the biomechanically corrected IOP (bIOP), the central corneal thickness (CCT), and six dynamic corneal response (DCR) parameters, namely DA, DARatio2mm, IIR, SP-A1, CBI, and SSI. Participants were divided into three groups according to the changes in patients’ bIOP after mydriasis.

**Results:** Intra-operator repeatability was generally high in most of the DCR parameters obtained before and after dilation. The mean changes in bIOP and CCT after dilation were −0.12 ± 1.36 mmHg and 1.95 ± 5.23 μm, respectively. Only ∆DARatio2mm, ∆IIR, and ∆CBI exhibited a statistically significant correlation with ∆CCT (*p* < 0.05). The changes in all DCR parameters, especially ∆DA and ∆SP-A1 were also correlated with ∆bIOP (*p* < 0.01)—a 1-mmHg change in bIOP was associated, on average, with 5.612 and −0.037 units of change in SP-A1 and DA, respectively. In contrast, the weakest correlation with ∆bIOP was exhibited by ∆SSI.

**Conclusion:** Most corneal DCR parameters, provided by the Corvis ST, were correlated with IOP, and more weakly with CCT. Changes experienced in CCT and IOP should therefore be considered in studies on corneal biomechanics and how it is affected by disease progression and surgical or medical procedures.

## Highlights

Most corneal biomechanical metrics, especially SP-A1 and DA provided by Corvis ST, proved to be correlated with IOP in a study of matched clinical data obtained before and after dilation.

## Introduction

To focus light rays on the retina, the cornea needs to remain transparent and maintain a suitable shape that stays stable with the diurnal changes in intraocular pressure (IOP). This is made possible by the cornea’s microstructure, which confers the tissue with complex biomechanical properties (Meek and Knupp, 2015). Corneal biomechanics has been a hot topic in ophthalmology research due to its prospective applications in the diagnosis, management and treatment of several clinical conditions, including keratoconus (Rabinowitz, 1998). Understanding corneal biomechanical properties is also of great importance in the planning of refractive surgery, where these properties can help identify patients at high risk of developing iatrogenic ectasia after laser vision correction (Esporcatte et al., 2020).

The *in vivo* evaluation of corneal biomechanical behavior has recently become possible through the introduction of the Ocular Response Analyzer (ORA) and Corvis ST (CVS). Of the two devices, Corvis ST provides more information on corneal biomechanical response, based on ultra-high-speed Scheimpflug technology which records the entire process of corneal deformation and provides measurements such as the stiffness parameter at first applanation (SP-A1) and the integrated inverse radius (IIR). While these biomechanical metrics have been shown to have strong correlation with the cornea’s overall stiffness, they are not independent parameters, but influenced by stiffness-unrelated events such as the diurnal variation in the IOP (Bao et al., 2015).

One of these events is mydriasis; an integral part of the pre-refractive surgery evaluation of patients. Qian et al., reported 35% of patients had a post-dilation variation in IOP of more than 2 mm Hg—31.1% of these patients experienced significant IOP increases after mydriasis, while the other 68.9% showed significant decreases (Qian et al., 2012). These changes may have had an effect on the Corvis ST biomechanical metrics. This study aims to identify the influence of changes in IOP caused by mydriasis on these metrics and evaluate the repeatability of these effects.

## Methods

### Study Design

The study followed the tenets of the Declaration of Helsinki and was approved by the Ethics Committee of the Eye Hospital, Wenzhou Medical University, China. The records of patients seeking myopic or astigmatic correction were examined at the corneal refractive surgery center and evaluated for inclusion in this study. The inclusion criteria were: the presence of myopia with astigmatism not exceeding 3.25 D, and with manifest spherical equivalent ≥ −10.00 D; and absence of ocular diseases (other than refractive errors). All 233 patients included underwent complete ophthalmic examination, and those wearing soft contact lenses were asked to suspend their use for 2 weeks prior to the examination. Informed consent was provided by all participants to use their data in research.

### Mydriasis Test

As part of the routine posterior segment examination, each participant received Mydrin-P (0.5% tropicamide and 0.5% phenylephrine hydrochloride; Santen Pharmaceuticals, Japan) three times (one drop each time) with 10 min between applications. The 233 participants included were divided into three groups (Group I, Group D, and Group S) according to the patients’ biomechanically corrected IOP (bIOP) value (obtained with the Corvis ST) after mydriasis test. If the bIOP value fell after the mydriasis test by more than (Sw_pre_ + Sw_pos_)/2 (where Sw_pre_ and Sw_pos_ meant the within-subject standard deviation before and after mydriasis test), the subject was included in Group D (47 subjects). If the bIOP value increased by more than (Sw_pre_ + Sw_pos_)/2 after the mydriasis test, the subject was included in Group I (33 subjects), and all remaining subjects were included in Group S (153 subjects).

### Biomechanical Evaluation

All Corvis ST (CVS) exams were taken in the sitting position, and all participants underwent measurements in a single session (≤2 h). CVS measurements were taken at two stages: pre- and post-dilation (before and after the mydriasis test, respectively). In each stage, measurements were repeated every 2 min until three satisfactory readings were obtained with the difference between the lowest and highest readings being ≤2 mmHg (i.e., a total of six readings per subject). The same experienced clinician (WC) took all measurements, using the same instruments throughout, to limit potential variability associated with either the instrument or the operator. The Corvis ST was calibrated by the clinic’s technician before the study was started.

Central corneal thickness (CCT) and six dynamic corneal response (DCR) parameters that have been consistently linked to corneal stiffness (Vinciguerra et al., 2016; Roberts et al., 2017) were recorded at both pre- and post-dilation stages from Corvis ST. These parameters included the deformation amplitude (DA), the ratio between DA values at the apex and 2 mm from the apex (DARatio2mm), and the integrated inverse radius (IIR, the integrated sum of inverse concave radius values between first and second applanation events). The stiffness parameter at first applanation (SP-A1) was also included, and represented the difference between the adjusted air puff pressure at first applanation (AdjAP1) and bIOP divided by the defection amplitude at the first applanation (A1DeflAmp) (Reinstein et al., 2013).

SP-A1 = (adjAP1—bIOP)/(A1DeflAmp) Equation 1.

Also included were the Corvis biomechanical index (CBI) and the stress-strain index (SSI) which was developed to evaluate the material stiffness of corneal tissue (Eliasy et al., 2019).

### Statistical Analyses

Repeatability of the three CVS measurements was assessed using the within-subject standard deviation (Sw), within-subject coefficient of variation (CoV) and intraclass correlation coefficient (ICC), and was used to evaluate the reliability of CVS measurements both before and after dilation. The average of the three readings considered in each stage was used for statistical analysis using SPSS (version 20.0, IBM, Inc.). The min sample size was calculated based on paired-sample t-tests with the help of G*Power (version 3.1.2, Franz Faul, University Kiel, Germany), and at least 30 participants were required for each subgroup. Paired-sample T tests analyzed the differences between pre- and post-dilation biometric values, while Groups I, D, and S were compared *via* One-way ANOVA. ∆ meant the differences between before and after mydriasis test. The relationship between ∆DA, ∆DARatio2mm, ∆IIR, ∆SP-A1, ∆CBI, ∆SSI, and differences in CCT or bIOP pre-versus post-dilation were assessed using Pearson’s or Spearman’s correlation factor for normally and non-normally distributed data, respectively. A stepwise approach to multiple linear regression analysis was used to identify associations between ∆CCT and ∆bIOP on one hand, and the changes in ocular biomechanical metrics pre- and post-dilation on the other. *p* values <0.05 were considered statistically significant.

## Results

The mean age of all participants was 26.3 ± 7.1 years and was not significantly different among the three study groups (*p* = 0.590). Corvis ST results before and after mydriasis, along with the repeatability measures, are displayed in Table 1. The data indicated that there was generally high intra-operator repeatability in most the DCR parameters recorded. For bIOP, within-subject standard deviation (Sw) was 0.93 mmHg before mydriasis and similar after (0.96 mmHg). The coefficient of variation (CoV) was approximately 6% (5.99% before mydriasis and 6.29% after). 20.2% of subjects showed an increase in bIOP measurement post-dilation (2.01 ± 1.50 mmHg, 1.00–9.37 mmHg, Group I), 14.2% exhibited a decrease post-dilation (−1.85 ± 0.80 mmHg, −0.97 ∼ −5.4 mmHg, Group D), and the remaining 65.6% had stable bIOP readings (−0.06 ± 0.50 mmHg, −0.93–0.93 mmHg, Group S).

**TABLE 1:** Repeatability of corvis ST parameters.

Metrics	Stages	Mean + Std	Sw	CoV	ICC
bIOP, mmHg	Pre	15.5 ± 2.2	0.93	5.99	0.835
	Pos	15.4 ± 2.1	0.96	6.29	0.818
CCT, μm	Pre	549.6 ± 33.8	5.15	0.94	0.977
	Pos	551.5 ± 33.5	5.05	0.92	0.978
DA, mm	Pre	1.04 ± 0.1	0.04	3.84	0.851
	Pos	1.04 ± 0.1	0.04	4.10	0.845
DARatio2mm	Pre	4.27 ± 0.4	0.14	3.23	0.879
	Pos	4.24 ± 0.39	0.16	3.75	0.849
IIR, mm^−1^	Pre	8.85 ± 0.98	0.41	4.68	0.827
	Pos	8.81 ± 0.98	0.38	4.26	0.864
SP-A1, mmHg/mm	Pre	106.60 ± 17.31	6.22	5.83	0.882
	Pos	105.99 ± 17.73	6.84	6.47	0.867
CBI	Pre	0.32 ± 0.23	0.08	-	0.887
	Pos	0.30 ± 0.23	0.08	-	0.899
SSI	Pre	0.90 ± 0.12	0.06	6.12	0.804
	Pos	0.90 ± 0.13	0.05	5.80	0.846

bIOP, biomechanically corrected IOP; CCT, central corneal thickness; DA, deformation amplitude; DARatio2mm, the ratio between DA values at the apex and 2 mm from apex; IIR, integrated inverse radius; SP-A1, the stiffness parameter at first applanation; CBI, corvis biomechanical index; SSI, stress-strain index; Pre, undilated pupil; Pos, after mydriasis test; Sw, within-subject standard deviation; CoV, coefficient of variation (%); ICC, intraclass correlation coefficient.

One of the parameters, CCT, showed excellent repeatability (ICC ≥ 0.90), while all others had good repeatability (ICC ≥ 0.75). Most DCR parameters presented CoV for repeatability below 5%. SP-A1 and SSI had slightly higher CoV (5.83%/6.47% and 6.12%/5.80%, respectively, before and after pupil dilation). As the mean CBI was very low (∼0.32), the CoV was not calculated. Repeatability of the biomechanical metrics was similar at the pre- and post-dilation stages.

**FIGURE 1:**
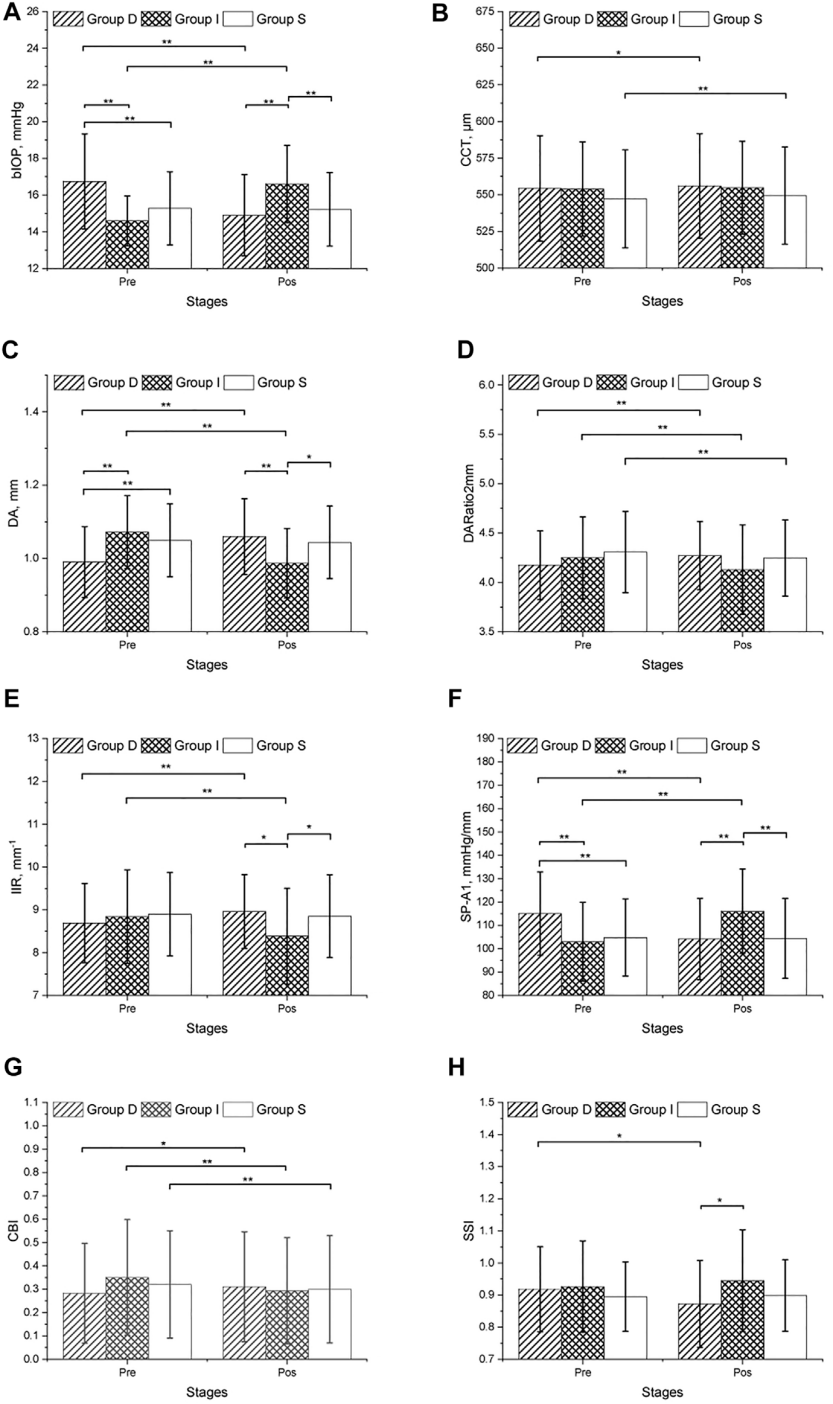
CVS metrics collected before and after the mydriasis test in three groups.

The CVS metrics collected before and after the mydriasis test are presented in Tables 1, 2; Figure 1. CCT, DARatio2mm, and CBI changed statistically significantly after mydriasis, while the other five DCR parameters remained stable. Before dilation, all metrics except bIOP, DA, and SP-A1 were similar across the three patient groups (*p* > 0.05), while statistically significant differences (*p* < 0.05) were observed in bIOP, DA, IIR, SP-A1, and SSI after dilation. bIOP, DA, IIR, and SP-A1 changed significantly after the mydriasis test in Groups D and I while remaining stable in Group S. DARatio2mm and CBI also changed significantly in all three groups after dilation. Further, the corresponding changes in CCT were significant in Groups D and S, while SSI only varied significantly in Group D.

**TABLE 2:** CVS metrics collected before and after the mydriasis test in three groups.

Metrics	Groups	Pre-dilation	Post-dilation	Difference	*p*
bIOP, mmHg	Group D	16.7 ± 2.6	14.9 ± 2.2	−1.8 ± 0.8	<0.001
	Group I	14.6 ± 1.4	16.6 ± 2.1	2.0 ± 1.5	<0.001
	Group S	15.3 ± 2.0	15.2 ± 2.0	−0.1 ± 0.5	0.169
	*p*	<0.001	0.001	<0.001	-
CCT, μm	Group D	554.4 ± 35.9	556.0 ± 35.7	1.7 ± 5.6	0.046
	Group I	553.9 ± 32.1	554.9 ± 31.7	1.0 ± 4.7	0.247
	Group S	547.2 ± 33.4	549.5 ± 33.2	2.2 ± 5.2	<0.001
	*p*	0.327	0.414	0.412	-
DA, mm	Group D	0.99 ± 0.10	1.06 ± 0.10	0.07 ± 0.03	<0.001
	Group I	1.07 ± 0.10	0.99 ± 0.09	−0.08 ± 0.06	<0.001
	Group S	1.05 ± 0.10	1.04 ± 0.10	−0.01 ± 0.04	0.069
	*p*	<0.001	0.004	<0.001	-
DARatio2mm	Group D	4.18 ± 0.35	4.27 ± 0.35	0.10 ± 0.19	0.001
	Group I	4.25 ± 0.41	4.13 ± 0.45	−0.12 ± 0.15	<0.001
	Group S	4.31 ± 0.41	4.25 ± 0.39	−0.06 ± 0.23	0.002
	*p*	0.134	0.223	<0.001	-
IIR, mm^−1^	Group D	8.69 ± 0.93	8.96 ± 0.86	0.27 ± 0.33	<0.001
	Group I	8.84 ± 1.09	8.38 ± 1.12	−0.46 ± 0.87	0.005
	Group S	8.90 ± 0.97	8.85 ± 0.97	−0.05 ± 0.51	0.267
	*p*	0.445	0.022	<0.001	-
SP-A1, mmHg/mm	Group D	115.02 ± 17.86	104.13 ± 17.45	−10.89 ± 4.64	<0.001
	Group I	102.97 ± 16.89	116.04 ± 18.08	13.06 ± 7.20	<0.001
	Group S	104.80 ± 16.51	104.39 ± 17.12	−0.41 ± 5.72	0.382
	*p*	0.001	0.002	<0.001	
CBI	Group D	0.28 ± 0.21	0.31 ± 0.24	0.03 ± 0.09	0.032
	Group I	0.35 ± 0.25	0.29 ± 0.23	−0.06 ± 0.07	<0.01
	Group S	0.32 ± 0.23	0.30 ± 0.23	−0.02 ± 0.08	0.002
	*p*	0.409	0.944	<0.001	-
SSI	Group D	0.92 ± 0.13	0.87 ± 0.14	−0.05 ± 0.05	<0.01
	Group I	0.93 ± 0.14	0.94 ± 0.16	0.02 ± 0.10	0.306
	Group S	0.89 ± 0.11	0.90 ± 0.11	0.00 ± 0.06	0.420
	*p*	0.262	0.038	<0.001	-

bIOP, biomechanically corrected IOP; CCT, central corneal thickness; DA, deformation amplitude; DARatio2mm, the ratio between DA values at the apex and 2 mm from apex; IIR, integrated inverse radius; SP-A1, the stiffness parameter at first applanation; CBI, corvis biomechanical index; SSI, stress-strain index.

**FIGURE 2:**
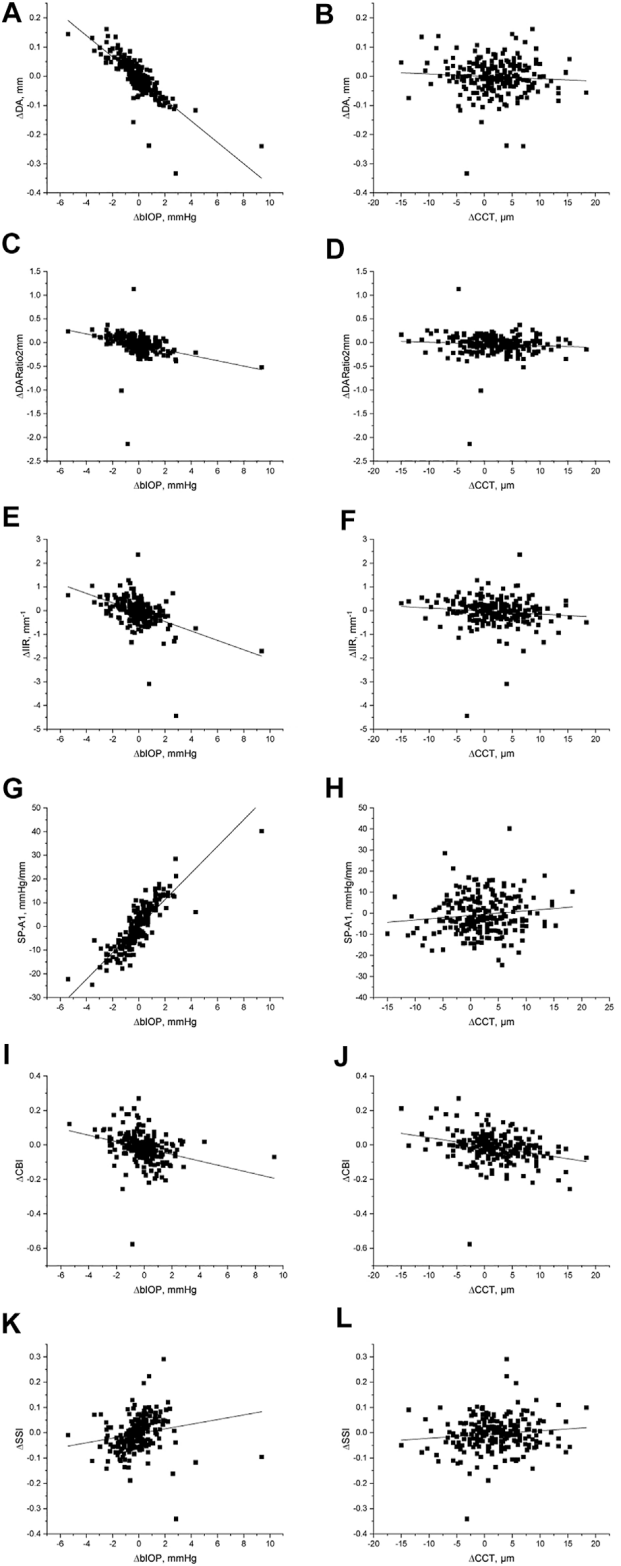
Scatter diagram and linear fit for the differences in DCR parameters recorded before and after the mydriasis test and the corresponding differences in bIOP and CCT.

In both pre and post dilation stages, all metrics except SSI were statistically different in male and female populations (all *p* < 0.05), but the differences in most metrics became non-significant (*p* > 0.05) after correction for CCT and bIOP. Changes in all DCR parameters were correlated with ∆bIOP (*p* < 0.01); only ∆DARatio2mm, ∆IIR and ∆CBI exhibited a correlation with ∆CCT (Table 3; Figure 2). ∆bIOP was most closely correlated with ∆DA and ∆SP-A1 (r = −0.857 and 0.856, respectively), and least correlated with ∆SSI (r = 0.414).

**TABLE 3:** Correlation between differences in DCR parameters recorded before and after the mydriasis test and the corresponding differences in bIOP and CCT.

**Dependent variables**	**∆bIOP, mmHg**		**∆CCT, μm**	
	*r*	*p*	*r*	*p*
∆DA, mm	−0.857	<0.001	−0.080	0.226
∆DARatio2mm	−0.524	<0.001	−0.154	0.019
∆IIR, mm^−1^	−0.479	<0.001	−0.192	0.003
SP-A1, mmHg/mm	0.856	<0.001	0.119	0.070
∆CBI	−0.423	<0.001	−0.288	<0.001
∆SSI	0.414	<0.001	0.087	0.186

∆, the difference before and after mydriasis; bIOP, biomechanically corrected IOP; CCT, central corneal thickness; DA, deformation amplitude; DARatio2mm, the ratio between DA values at the apex and 2 mm from apex; IIR, integrated inverse radius; SP-A1, the stiffness parameter at first applanation; CBI, Corvis biomechanical index; SSI, stress-strain index; Pre, undilated pupil; Pos, after mydriasis test; Sw, within-subject standard deviation; CoV, coefficient of variation (%); ICC, intraclass correlation coefficient.

In addition, multiple linear regression analyses were conducted (see Table 4). Correlation between ∆bIOP and the six DCR parameters was analyzed based on a second-order polynomial regression. ∆DA and ∆SP-A1 were highly correlated with ∆bIOP (R^2^ = 0.677 and 0.737, respectively), while there was minimal correlation between ∆bIOP and each of ∆CBI and ∆SSI (R^2^ = 0.173 and 0.033, respectively). Results of the analysis indicated that a 1-mmHg change in bIOP was associated with -0.037, −0.056, −0.198, and 5.612 units of change in DA, DARatio2mm, IIR, and SP-A1, respectively.

**TABLE 4:** Multiple linear regression analysis results between of correlation between ∆bIOP, ∆CCT, and six DCR parameters.

**Dependent variables**	**Parameters**	**Β**	***p*** **value**	**Regression equation**	**Adjusted ** ***R*** ^**2**^	**F** ^**b**^	***p*** ** value**
∆DA	∆bIOP	−0.824	<0.001	∆DA (mm) = −0.037 × ∆bIOP (mmHg) − 0.002 (mm)	0.677	487.409	0.000
∆DARatio2mm	∆bIOP	−0.344	<0.001	∆ DA Ratio 2 mm (mm) = −0.056 × ∆bIOP (mmHg) − 0.043 (mm)	0.114	30.919	0.000
∆IIR	∆bIOP	−0.462	<0.001	∆ IIR (mm) = −0.198 × ∆bIOP (mmHg) − 0.013 × ∆CCT − 0.040 (mm)	0.220	33.738	0.000
	∆CCT	−0.115	0.049				
∆SP-A1	∆bIOP	0.850	<0.001	∆ SP-A1 (mmHg) = 5.612 × ∆bIOP (mmHg) + 0.126 × ∆CCT −0.341 (mmHg)	0.737	326.045	0.000
	∆CCT	0.125	<0.001				
∆CBI	∆bIOP	−0.298	<0.001	∆ CBI (mmHg) = −0.019 × ∆bIOP (mmHg) − 0.005 × ∆CCT − 0.009 (mmHg)	0.173	25.225	0.000
	∆CCT	−0.302	<0.001				
∆SSI	∆bIOP	0.192	0.003	∆ SSI (mmHg) = 0.009 × ∆bIOP − 0.003 (mmHg)	0.033	8.838	0.003

## Discussion

Corneal biomechanics is a subject of tremendous clinical research interest in modern ophthalmology. Knowledge of corneal biomechanics is useful in several clinical applications, including management of glaucoma and ectasia risk profiling (Esporcatte et al., 2020; Zimprich et al., 2020). The integration of tomographic and biomechanical data has demonstrated potential to improve the accuracy of detection of ectatic disease and identify susceptibility to develop this complication after laser vision correction (Ambrósio et al., 2017). Despite substantial developments over the last 2 decades, *in vivo* characterization of dynamic corneal biomechanical response remains influenced by IOP, as observed by Bao et al. (2015) and Roberts (2014). This new study uses the IOP changes observed after pupil dilation to assess the effects of these changes on the corneal biomechanical metrics provided by the Corvis ST.

Previous studies showed that most corneal biomechanical metrics provided by ORA and Corvis ST were related to IOP and CCT (Roberts, 2014; Bao et al., 2015). In the present study, it was observed that DA, DARatio2mm, IIR, and SP-A1 may be correlated with the overall stiffness of the cornea, which is dependent on CCT but also directly influenced by IOP. Meanwhile, we have confirmed that these DCR parameters are influenced by the biomechanically corrected IOP to different extents. Our results showed that a 1-mmHg change in bIOP can induce −0.037, −0.056, −0.198, and 5.612 units of change in DA, DARatio2mm, IIR, and SP-A1, respectively, and that the correlation between each of these metrics and bIOP was significant. Likewise, SSI, which was designed to represent the tissue’s material stiffness and was intended to be independent of IOP (Eliasy et al., 2019), was also dependent on IOP, although to a lesser extent than other parameters.

Previous reports of these relationship were mixed, the DA was correlated with CCT and IOP (Hon and Lam, 2013; Ali et al., 2014; Huseynova et al., 2014), the DARatio2mm had high correlation with CCT and IOP (Vinciguerra et al., 2016), and the IIR correlated with CCT (Vinciguerra et al., 2016) while not with IOP. In other studies, DA, DARatio2mm, IIR, and SP-A1 were all correlated with CCT and IOP (Sedaghat et al., 2020), CBI was correlated with IOP and CCT (Yang et al., 2019), and SSI was correlated with IOP but not with CCT (Liu et al., 2020). The studies noted above were cross-sectional, and the variation range in both subjects and biometric parameters was large, which may have influenced the correlation results. In our longitudinal study, the changes in corneal biomechanical metrics caused, in the same patient, by dilation were used to evaluate the relationships between the DCR parameters and both CCT and IOP. The study was designed as self-matched (between a short period before and after dilation) to exclude the effects of potential confounding factors such as race, gender, age, CCT and IOP.

Recently, SP-A1, was introduced as a clinical metric with strong association with corneal stiffness. Considerable discussion has ensued in the literature around this novel parameter and its application in the diagnosis of corneal diseases like keratoconus (Yang et al., 2019). In this study, a strong positive correlation (r = 0.856, *p* < 0.001) was observed between the changes in SP-A1 and those in bIOP associated with dilation, suggesting that an increase in IOP would induce higher corneal stiffness. This finding has important clinical applications, for instance in detecting subclinical keratoconus in those due to undergo refractive surgeries. The difference in the Stiffness Parameter, SP-A1, between subclinical keratoconus and healthy eyes is within 15–20 (Koc et al., 2019; Ren et al., 2021), a gap which can be covered by an IOP change of 3–4 mmHg. Therefore, a higher bIOP may influence the effectiveness of SP-A1 in detecting keratoconus.

Although our results showed that all six dynamic corneal response parameters were dependent on bIOP. This is compatible with research done by Herber et al., who found that CBI were minimally influenced by bIOP (Herber et al., 2019). And contradicted with earlier statement in which SSI was reported to be independent on IOP (Eliasy et al., 2019). The limited correlation between CBI and SSI on one hand, and bIOP on the other, suggests the potential of these novel parameters for use in clinical practice.

Some studies have shown that IOP fluctuates during the 24-h cycle. Chun et al. confirmed that while IOP fluctuations can vary, phasing over a 24-h period is relatively consistent (Zhu et al., 2020), and others have reported that IOP fluctuation was between 3 and 5 mmHg in healthy subjects (Luebke et al., 2019). The dependence of DCR parameters on IOP can then lead to changes in these metrics with the IOP diurnal fluctuation affecting their ability to detect corneal diseases such as keratoconus. Precautions were therefore taken to limit the possible influence of IOP diurnal fluctuations in this study, including ensuring that all exams were performed within a 2-h time period. The strong repeatability of the readings and the relatively low CoV for CCT suggested the limited impact of diurnal fluctuations both before and after mydriasis.

The components of Mydrin-P (tropicamide and phenylephrine hydrochloride) have been shown to cause increases in CCT although the underlying mechanism is unclear (Gao et al., 2006; Zeng and Gao, 2017). On the other hand, changes in IOP with pupil dilation may come from different sources. First, Phenylephrine can cause an increase in arterial blood pressure (Stavert et al., 2015), possibly leading to a similar effect in IOP. Second, pupil dilation may induce pigment liberation and the subsequent obstruction of the trabecular meshwork, which may hinder aqueous outflow and increase IOP (Kristensen, 1965). In contrast, uveoscleral outflow facilitation and inflow decrease can also occur after dilation, leading to a decrease in IOP (Valle, 1974; Tan et al., 2009). These conflicting factors can therefore cause an increase or a decrease in IOP as has been observed in our study.

One limitation of the study is the potential, unquantified pharmacologic effect of mydriasis on corneal microstructure and consequently corneal biomechanical properties. However, the small change in CCT after mydriasis (1.95 ± 5.23 μm) indicated that the possible effect of mydriasis may have been limited, and further study is required to consider the role of patient-specific factors in these relationships.

In conclusion, most corneal DCR parameters have been shown to be strongly related to IOP. This study focused on the correlation of biomechanical parameters and IOP and analyzed the repeatability of CVS measurement. The data collected confirmed our group’s previous conclusions, that comparing research groups based on Corvis ST with different IOPs and CCTs may lead to possible misinterpretations if both or one of which are not considered in the analysis; the results also validate the stability of several new corneal parameters. Importantly, most parameters provided by Corvis ST, including some stiffness parameters, are influenced by IOP.

## Data Availability

The original contributions presented in the study are included in the article/Supplementary Material, further inquiries can be directed to the corresponding authors.
